# Operational Impact of Redirection From the Pediatric Emergency Department: A Matched Cross‐Sectional Study

**DOI:** 10.1111/acem.70151

**Published:** 2025-09-19

**Authors:** Erica Qureshi, Brett Burstein, Kelly Cummins, Garth Meckler, Jessica Moe, Steven P. Miller, Quynh Doan

**Affiliations:** ^1^ Faculty of Medicine University of British Columbia Vancouver British Columbia Canada; ^2^ British Columbia Children's Hospital Research Institute Vancouver British Columbia Canada; ^3^ Montreal Children's Hospital, Division of Pediatric Emergency Medicine McGill University Health Centre Montréal Quebec Canada; ^4^ Department of Biostatistics, Epidemiology and Occupational Health McGill University Montreal Quebec Canada; ^5^ Department of Pediatrics University of British Columbia Vancouver British Columbia Canada; ^6^ Department of Emergency Medicine University of British Columbia Vancouver British Columbia Canada; ^7^ BC Centre for Disease Control Vancouver British Columbia Canada; ^8^ Department of Emergency Medicine Vancouver General Hospital Vancouver British Columbia Canada; ^9^ Department of Emergency Medicine BC Children's Hospital Vancouver British Columbia Canada

## Abstract

**Background:**

Programs redirecting patients with non‐urgent presentations from Emergency Departments (EDs) to the community (ED2C), by providing them a booked community appointment in lieu of waiting for ED care, may reduce ED crowding. We sought to evaluate the department‐ and patient‐level impact of an ED2C program in an urban tertiary pediatric ED.

**Methods:**

We conducted a matched cross‐sectional study to describe patients redirected by a pediatric ED2C program and determine if the program changed ED operations. Days with the program were matched on day type (weekday vs. weekend) and department volume (±10%) to days when ED patients were not being redirected. Measures of ED flow and utilization on days with and without the program were compared using *t*‐tests and linear regression models.

**Results:**

Of the 6164 patients eligible for the ED2C program for 53 days that redirection was offered, 900 were redirected (14.6%). On average, 17.7 (SD 8.5) patients were redirected and 92.4 (SD 23.7) eligible patients were not redirected each day the ED2C was in operation. Patients who were redirected had a significantly shorter length of stay (LOS) than those who were eligible but not redirected (2.9 ± 2.0 h vs. 8.5 ± 4.3 h, *p*‐value < 0.0001). Three patients who were redirected (0.3%) and 11 eligible but not redirected (0.2%) returned to the ED and were hospitalized. Average median departmental LOS, time to physician assessment, daily proportion hospitalized patients, proportion of patients left without being seen, and ED return visits did not differ on days with and without the program.

**Conclusions:**

A small proportion of eligible patients were redirected. These patients experienced a lower LOS, without increasing the proportion of return visits. ED operations were unchanged. Refining eligibility criteria for pediatric redirection with an emphasis on patient safety is necessary.

## 1 Introduction

Reducing crowding in the Emergency Department (ED) is a complex but important goal due to the negative impacts of crowding on both individual patients and ED operations. Crowding within the ED impacts the timeliness and safety of care and is associated with unnecessary tests [1], increased wait times [2], decreased patient satisfaction [3, 4], and an increase in the proportion of patients who ‘leave without being seen’ (LWBS) [5]. Crowding in pediatric EDs is driven by input factors including a high volume of non‐urgent visits. Many of these visits can effectively be handled by community healthcare resources as they are for non‐urgent healthcare concerns [2, 6, 7, 8]. Strategies that reduce the impact of non‐urgent visits may reduce crowding and thereby improve pediatric ED efficiency.

Various strategies have been described to reduce the impact of non‐urgent visits [9]. One strategy that holds promise connects patients with non‐urgent presentations to community locations for care through “Emergency Department to Community (ED2C)” redirection programs. ED2C programs redirect ED patients with non‐urgent healthcare needs to a community healthcare provider at a scheduled appointment in lieu of waiting for care within the ED. These programs provide needed reassurance to patients seeking care, provide an alternative to ED care, and facilitate healthcare navigation. ED2C programs may decrease the impact of low‐acuity visits and enhance ED operations and flow by redirecting visits that are non‐urgent.

In 2022, the pediatric healthcare system faced unprecedented volume as Respiratory Syncytial Virus, Influenza, and COVID‐19 infections converged [10]. Given the surge in pediatric ED volume and the crowding that resulted, a Canadian urban tertiary pediatric ED launched an ED2C program and identified children as eligible for redirection based on their presenting problem and level of triage acuity. Data regarding the operational impact of such programs is lacking, and the patient‐ and ED‐level impacts of such a program remain unclear. This study sought to (1) describe the population eligible for and redirected by the ED2C program and, (2) determine if the program impacted ED operations and resulted in changes in measures of ED flow and utilization.

## 2 Methods

### 2.1 Study Design

We conducted a matched cross‐sectional study using administrative data from an urban tertiary pediatric ED in Canada that serves children less than 18 years of age. Ethics approval was obtained from the research institute of The McGill University Health Centre.

### 2.2 Intervention

The ED2C program was made possible due to an existing network of 12 clinics located surrounding the pediatric ED that were interested and able to receive redirected patients, and a provincial digital platform with real‐time information about appointment availability. Community clinics included both urgent care–style and family practice settings staffed by physicians. Eligibility was determined by assessing the child's Pediatric Canadian Triage and Acuity Scale (CTAS) assignment and reason for visit. The Pediatric CTAS is a 5‐level acuity triage system used in Canadian pediatric EDs (level 1 Resuscitation; level 2 Emergent; level 3 Urgent; level 4 Less Urgent; and, level 5 Non‐Urgent) [11]. Children were eligible for the ED2C program if they were assigned CTAS 4 or 5 and if they were not seeking care for a second opinion, a behavioral or mental health concern, or an injury.

ED2C facilitators were hired from existing pediatric ED nurses and allied health professionals (e.g., nutritionists, physiotherapists, child life specialists). Facilitators were trained by the pediatric ED nurse manager—who oversaw the implementation and operations of the ED2C program—via dedicated sessions and supporting documentation. In general, ED2C facilitators were present from 07:00 to 23:00 (split between a daytime and an evening shift). During shifts, facilitators reviewed triage‐identified eligible patients and approached them individually. Although facilitators aimed to offer redirection to all eligible patients, they independently prioritized whom to approach based on workflow demands; detailed documentation of this decision‐making process was not captured. Redirection conversations occurred in a designated pediatric ED room, ensuring privacy while offering community appointments. Families could select appointment times based on clinic proximity and availability. No transportation assistance was provided.

### 2.3 Selecting and Summarizing Program and Program‐Free Days

ED2C facilitators operated the program between October 25th and December 18th, 2022. Days with the ED2C program were matched to days without the program. Each program day was matched to one program‐free day from outside the intervention period (September 22, 2022 to March 19, 2023). Matches were based on day type (weekday: Monday to Thursday, or weekend: Friday to Sunday), and 24‐h department volume (±10%). When multiple matches were available, the program‐free day closest in time to the program day was selected.

After matching, variables for program and program‐free days were summarized at the day level. Program days were defined as the 16‐h period beginning at the start of an ED2C Facilitator shift. This length was selected to eliminate overlap between consecutive program days while acknowledging that the ED2C program may have downstream effects on patient flow stretching beyond the period when the ED2C Facilitator was working. The same 16‐h period was used when summarizing program‐free days.

### 2.4 Measurements

We measured the proportion of patients who were eligible for the ED2C program, and among those eligible, the proportion who were redirected.

Program impact on ED operations was assessed by considering measures of flow and utilization. Outcome measures of interest included median departmental length of stay (LOS), median departmental wait time to the physician assessment, the proportion of patients who ‘left without being seen’ (LWBS), the proportion of patients admitted to hospital, and the proportion of visits that returned to the same ED within 3‐days and within 7‐days. Individual LOS was defined by the time from registration to disposition from the ED (including those who LWBS and those who accepted the ED2C program). Wait time to the physician assessment was defined as the time from registration to first assessment with a physician in the ED. The proportion of daily visits LWBS was defined as the number of patients who registered in the ED and were not redirected but left prior to physician assessment. Any patient who returned to the same pediatric ED following their initial visit within 3 or 7 calendar days was considered as having a return visit.

We used descriptive statistics (mean ± SD) to summarize patient age, sex, reason for visit, visit acuity, and disposition location. We used the reason for the visit captured by the triage nurse to determine if a patient was eligible for the program. Visit acuity was based on the CTAS assignment. Return visits to the ED, which may indicate disease progression or dissatisfaction with their original visit or with the ED2C program, were identified through patients' unique identification numbers.

### 2.5 Data Analysis

We considered all visits to the pediatric ED during the ED2C program period. Descriptive statistics were used to summarize the population eligible for the ED2C program and the subset who were redirected by the ED2C program. We compared LOS, 3‐day and 7‐day return visits to the ED between those who were eligible for the program and redirected and those who were eligible for the program but not redirected using 2‐tailed paired *t*‐tests.

To identify differences between each measure of ED flow and utilization on days with and without the ED2C program, we completed 2‐tailed paired *t*‐tests. To control for possible confounders, we also created a linear regression model for each outcome of interest and included the presence of the ED2C program as a dichotomous predictor. Models were adjusted for median age, average daily proportion of visits CTAS 1 to 3, and average daily proportion of visits CTAS 4 to 5.

If disposition location or CTAS assignment was missing, it was assumed that the individual was eligible for the ED2C program but did not accept the intervention. By handling missing data in this way, we did not erroneously overestimate the proportion of patients eligible for or redirected by the ED2C program. If missing data meant that the LOS or time to physician assessment could not be determined, then the visit was not included in the calculation of median daily departmental LOS or median time to physician assessment, but the visit contributed to the daily volume.

### 2.6 Sample Size

The sample size was fixed based on the number of days the ED2C program operated. However, we completed sample size calculations for each ED flow and utilization outcome of interest to ensure that our study would be adequately powered to detect a meaningful difference in our outcomes of interest between program and program‐free days. With 90% confidence, a sample of 44 matched program and program‐free days was required to estimate at least a: 30‐min difference in median daily LOS, a 1% difference in the proportion of daily return visits, a 1% difference in the proportion of daily visits LWBS, and a 5% difference in the proportion of daily visits admitted to hospital. Since the program ran for 53 days, our study was adequately powered.

## 3 Results

During the 53 days when the ED2C program operated, there were 13,689 ED visits, including 6164 (45%) visits eligible for redirection through the ED2C program.

Among eligible visits, 900 (14.6%) were offered the ED2C program and accepted redirection—this was 6.6% of all visits to the ED during the program period. On average, 17.7 (SD 8.5) patients were redirected and 92.4 (SD 23.7) eligible patients were not redirected each day the ED2C program was in operation. Eligible patients who were not redirected included those who were not offered redirection, were offered and declined redirection, or were interested in redirection but there was not a community appointment available at a preferred time and/or location. The nurse manager was not informed of any families who were upset about being offered redirection. Details regarding eligible patients who were offered and accepted redirection, those who were not redirected by the program, and days with and without the ED2C program are summarized in Table 1.

**TABLE 1:** Comparing differences in day‐level characteristics and measures of ED flow and utilization on days with the ED2C program, matched control days without the ED2C program, and for eligible patients based on redirection status.

	Patient Level—eligible patients on days with the ED2C program		Department level	
	Offered and accepted redirection (*n* = 900) Mean (SD)	Not redirected (*n* = 5264) Mean (SD)	Days with ED2C program Mean (SD)	Days without ED2C program Mean (SD)
Day‐level characteristics				
Median age (years)	4.3 (1.7)	3.9 (0.9)	4.2 (0.7)	3.7 (0.8)
Volume	17.7 (8.5)	92.4 (23.7)	264.3 (56.7)	262.3 (56.8)
Daily proportion CTAS 1–3	—	—	42.6 (5.0)	42.9 (4.6)
Daily proportion CTAS 4–5	100	100	57.3 (5.0)	57.1 (4.6)
Outcomes of interest				
Median departmental LOS (hours)	**2.9 (2.0)**	**8.5 (4.3)** ^a^	7.7 (2.9)	7.6 (2.7)
Median departmental time to physician assessment (hours)	—	—	2.6 (0.8)	2.5 (0.7)
Proportion of daily visits LWBS	—	—	18.8 (11.4)	18.8 (10.1)
Proportion of daily visits admitted to hospital	—	—	7.2 (1.6)	6.8 (1.7)
Proportion of return visits within 3‐days	3.8 (5.2)	5.3 (2.7)	5.1 (1.3)	5.0 (1.7)
Proportion of return visits within 7‐days	6.3 (8.9)	7.8 (3.1)	7.6 (1.4)	7.1 (2.0)

^a^ 2‐tailed *t*‐test, *p*‐value < 0.0001.

The ED LOS was lower among redirected patients (2.9 ± 2.0 h) compared to those who were not redirected (8.5 ± 4.3 h), *p*‐value < 0.0001. Among these patients, the proportion of return visits within 3 days was 3.8% (SD 5.2), and 5.3% (SD 2.7) for patients who were eligible but not redirected (*p*‐value = 0.07). The proportion of 7‐day return visits was 6.3% (SD 8.9) among those who were offered and accepted redirection and was 7.8% (SD 3.1) for those who were not redirected (*p*‐value = 0.25).

With respect to the characteristics of patients eligible for the ED2C program, we did not observe statistically significant differences in median patient age or sex between those who were, or were not, redirected through the ED2C program. These characteristics are summarized in Table 2. Respiratory illness (with or without fever) was the most common reason for visit, regardless of redirection status. Considering those redirected by the ED2C program, 92.3% (*n* = 831) were visiting the emergency department with respiratory illness (with or without fever).

**TABLE 2:** Characteristics of patients eligible for the ED2C program.

	Redirection offered and accepted (*n* = 900)	Not redirected (*n* = 5264)
	*n* (%)	*n* (%)
Sex		
Female	441 (49)	2485 (47.2)
Male	459 (51)	2779 (52.8)
CTAS		
4 – less urgent	197 (21.9)	2983 (56.7)
5 – non‐urgent	703 (78.1)	2281 (43.3)
Reason for Visit		
Respiratory illness (with or without fever)	831 (92.3)	4243 (80.6)
Gastrointestinal tract problems	31 (3.4)	489 (9.3)
Altered level of consciousness	9 (1.0)	162 (3.1)
Dermatologic problems	7 (0.8)	85 (1.6)
Other	22 (2.4)	280 (5.3)
Missing	0	5 (0.09)

Among the 5264 patients who were eligible for but not redirected by the program, 1872 patients LWBS (35.6%) and 32 were admitted to hospital (0.6%). Among those admitted to hospital, 28 were assigned CTAS 4 and four were assigned CTAS 5 (results not shown in table).

Three patients who were offered and accepted redirection (one assigned CTAS 4 and two assigned CTAS 5 on their initial visit) returned to the ED and were admitted to hospital on their return visit (0.3%). One of these patients was assigned CTAS 5 on their initial visit and was assigned CTAS 1 on their return visit within 3 days. Additional details about patients who were eligble for the ED2C program who returned to the ED and were admitted on their return visit can be found in Table S1.

All program days could be matched to a non‐program day. There were no statistically significant differences in median patient age, daily volume, or the proportion of visits assigned CTAS 1–3 or CTAS 4–5 between days with the ED2C program and their matched controls. Overall, we found no difference in our outcome measures of interest when comparing days with and without the ED2C program. Adjusting for daily age, average proportion of daily visits CTAS 1–3, and average proportion of daily visits CTAS 4–5, the linear regression models also found no association between the ED2C program and our outcomes of interest (Table 3).

**TABLE 3:** Impact of ED2C program on each department‐level outcomes of interest controlling for median age, average daily proportion of visits CTAS 1–3, and average daily proportion of visits CTAS 4–5.

Department level outcomes of interest	aOR (95% CI)	*p*
Median departmental LOS (hours)	0.3 (−0.9, 1.4)	0.67
Median departmental time to physician assessment (hours)	0.2 (−0.1, 0.5)	0.28
Proportion of daily visits LWBS	0.9 (−3.5, 5.3)	0.68
Proportion of daily visits admitted to hospital	0.2 (−0.4, 0.8)	0.50
Proportion of return visits within 3‐days	0.4 (−0.3, 1.1)	0.23
Proportion of return visits within 7‐days	0.5 (−0.1, 1.2)	0.09

Abbreviation: aOR, Adjusted odds ratio.

## 4 Discussion

The ED2C program redirected an average of 17.7 patients each day it operated, the majority of whom had respiratory illness (with or without fever). While the ED2C program reduced the LOS for redirected patients, the proportion of patients redirected was low; only 6.6% of the total volume visiting the ED during the program period and 14.6% of eligible patients were redirected. As such, we did not observe changes in departmental operations such as LOS, time to physician assessment, the proportion of visits admitted to hospital, LWBS, and return visits on days with and without the ED2C program.

Efforts to reduce pediatric ED crowding often rely on families recognizing non‐urgent concerns and their ability to access alternate locations for care. The ED2C program offers a different pathway within the ED, allowing reassurance and supporting healthcare navigation. A previous study transferring non‐urgent patients to their primary care provider demonstrated a lower proportion of return visits, decreased pediatric ED LOS, and reduced healthcare costs [12]. Our findings align with this work, showing reduced LOS along with patients who accepted redirection, and extend it by demonstrating that this is achievable when patients are redirected to a community provider even if it is not their own primary care provider.

Despite this, the low proportion of eligible patients that were redirected limited the program's impact on departmental operations. A survey of parents waiting in a pediatric ED found almost 60% would be interested in accepting a community healthcare appointment in lieu of waiting in the ED if it was available [13]. Nevertheless, only 14.6% of eligible patients were redirected in this study. On average, 264.3 patients visited the ED on program days (SD 56.7), with 92.4 eligible for redirection but not redirected. Therefore, there is a large opportunity to expand the proportion of patients redirected by a pediatric ED2C program, which may decrease crowding and improve ED operations. It is unclear what volume needs to be redirected from the pediatric ED for operations to be improved. Simulation studies in general EDs suggest improvements in departmental operations result when 20% of visits are redirected [14]. However, redirecting just 8% of visits from a general ED was associated with a significant decrease in time to physician assessment and the proportion of visits that LWBS [15]. Sustained evaluation of pediatric ED2C programs is critical to ascertain the redirection rates required to see associated improvements in pediatric ED operations.

Importantly, while ED operations were the focus of this evaluation, the benefits of redirection may extend beyond the department. For example, the care experience for patients may be improved; a redirection program in a general ED found over 80% of patients redirected were satisfied with community care [16]. Redirection also offers opportunities for vaccinations and well‐child care that may otherwise be missed [17]. Local legal and economic considerations may influence the feasibility and implementation of redirection programs. Additional research is needed to explore additional benefits of redirection and to determine barriers and facilitators to redirection such that ED2C programs can be expanded to more eligible patients while simultaneously better targeting those who are likely to accept redirection. In doing so, pediatric ED leadership can make informed choices about the departmental resources required to implement and sustain redirection and the benefits that are associated with ED2C programs.

While the ED2C program did not improve department operations, our findings that pediatric ED operations were not negatively impacted and remained relatively stable is important. For example, our finding that LOS and time to physician assessment were unchanged on days with and without the ED2C program provides evidence that providing redirection does not slow down flow through the ED. Further, we found that the proportion of visits that LWBS was unchanged on days with and without the ED2C program, which may suggest that redirection conversations do not unknowingly empower patients to LWBS once they are provided reassurance that they may be safe to delay care. Further, some may worry that those redirected from the ED to community locations for care may have a higher proportion of return visits—either because they are dissatisfied with community care or their condition deteriorates. Our finding that the proportion of return visits does not differ, with a marginally significant trend that those redirected have a lower proportion of return visits, provides evidence that patients with non‐urgent presentations can be successfully redirected to community for care.

Defining who should be eligible for pediatric redirection is complex. A review of strategies to reduce the impact of non‐urgent visits on the pediatric ED found that triage assignment was the indicator of how quickly patients need to be treated within the ED, not if a child is safe to leave it. One study using non‐urgent triage scores successfully redirected pediatric patients, with only 7% returning to the pediatric ED and none requiring admission [18]. In contrast, our results highlight potential risks with this approach: three patients who were redirected by the ED2C program returned to the ED and were admitted to hospital, one of whom was assigned a CTAS score of 1 on their return visit. While this represents only 0.3% of redirected patients—and 0.2% of eligible but not redirected patients were also admitted after a return visit—such events can undermine providers' perceptions of program safety and program acceptability. Similarly, other interventions using nurse assessment for redirection also had redirected patients return and require hospital admission [19]. Though infrequent, these outcomes may shape how caregivers and providers perceive the safety and acceptability of redirection. Overall, these findings suggest that triage may not be a sufficient criterion for pediatric redirection.

### 4.1 Limitations

ED2C Facilitators did not consistently keep a record of which eligible patients were offered redirection through the ED2C program. As a result, it is not possible to know how many patients were approached and declined redirection, how many were interested but there was not a preferable community appointment available, or how many eligible patients were not offered the ED2C program. Future research should capture redirection conversations and their outcomes to better characterize the feasibility of ED2C programs including barriers and facilitators to redirection.

We were also unaware if a redirected patient attended their community appointment or the clinical outcomes for this group. Understanding these factors is important for future research such that the patient‐level impact of an ED2C program can be fully ascertained.

Further, as we were limited to retrospective administrative data, we were unable to determine if return visits were for the same or different reasons. To overcome this, we considered any return visit to the pediatric ED, regardless of the reason for visit, as a true return visit (i.e., for the same reason as the initial visit). Therefore, our reported proportions of return visits to the ED may be overestimations of the true proportion as some return visits may be for reasons unrelated to their initial ED visit.

## 5 Conclusions

We found that a small proportion of patients eligible for the ED2C program were redirected. While these patients experienced a significantly lower LOS, the proportion of eligible patients who are redirected may need to be increased for a pediatric ED2C program to impact ED operations. Additionally, refining eligibility criteria for pediatric redirection with an emphasis on patient safety and acceptability is necessary.

## Author Contributions

Study concept and design: all authors. Acquisition of the data: Erica Qureshi, Brett Burstein, Kelly Cummins, Quynh Doan. Analysis and interpretation of the data: Erica Qureshi, Brett Burstein, Quynh Doan. Drafting of the manuscript: all authors. Critical revision of the manuscript: all authors. Statistical expertise: Erica Qureshi, Brett Burstein, Quynh Doan, consultation with Melissa Braschel from the Clinical Research Support Unit at BC Children's Hospital Research Institute (recognized in acknowledgements). Acquisition of funding: Not applicable.

## Conflicts of Interest

The authors declare no conflicts of interest.

## Supporting information


**Table S1:** Information about patients who were eligible for the ED2C program and then returned to the ED and were admitted to hospital on their return visit.

## Data Availability

The data that support the findings of this study are available on request from the corresponding author. The data are not publicly available due to privacy or ethical restrictions.
