# Supplementary material for: Operational Impact of Redirection From the Pediatric Emergency Department: A Matched Cross‐Sectional Study
Source: Acad Emerg Med. 2025 Sep 19;32(12):1320–6. doi: 10.1111/acem.70151 (PMC12690234; doi:10.1111/acem.70151)
Supplement: Supplementary file 1 — Table S1: Information about patients who were eligible for the ED2C program and then returned to the ED and were admitted to hospital on their return visit. [file ACEM-32-1320-s001.docx]

**Supplementary Table 1: Information about patients who were eligible for the ED2C program and then returned to the ED and were admitted to hospital on their return visit**

| **Initial Visit Disposition Location** | **Age (years)** | **Sex** | **Initial Visit CTAS** | **Reason for initial visit** | **Returned to the ED within** | **Return visit CTAS** | **Reason for return visit** |
| --- | --- | --- | --- | --- | --- | --- | --- |
| **Redirected** | 3 | Female | 5 | Exposure to contagious illness | 7-days | 3 | Dyspnea |
|  | 16 | Male | 4 | Headache | 7-days | 2 | Headache |
|  | 1 | Male | 5 | Exposure to contagious illness | 3-days | 1 | Dyspnea |
| **Eligible, not redirected*** | 2 | Male | 5 | Exposure to contagious illness | 3-days | 3 | Joint swelling |
|  | 1 | Male | 4 | Exposure to contagious illness | 3-days | 2 | Cough and fever |
|  | 15 | Female | 4 | Abdominal Pain | 3-days | 3 | Exposure to contagious illness |
|  | 13 | Male | 4 | Abdominal Pain | 3-days | 3 | Abdominal pain |
|  | 1 | Male | 4 | Dyspnea | 7-days | 2 | Dyspnea |
|  | <1 | Male | 4 | Fever | 3-days | 3 | Fever |
|  | 17 | Female | 4 | Abdominal Pain | 3-days | 4 | Abdominal Pain |
|  | 5 | Female | 4 | Fever | 3-days | 3 | Altered level of consciousness |
|  | 4 | Male | 4 | Fever | 3-days | 4 | Fever |
|  | 4 | Male | 4 | Abdominal Pain | 3-days | 3 | Abdominal pain |
|  | 4 | Female | 4 | Exposure to contagious illness | 3-days | 2 | Altered level of consciousness |
